# Coronary sinus electrogram characteristics predict termination of AF with ablation and long‐term clinical outcome

**DOI:** 10.1111/jce.15618

**Published:** 2022-07-28

**Authors:** Shohreh Honarbakhsh, Richard J. Schilling, Emily Keating, Malcolm Finlay, Ross J. Hunter

**Affiliations:** ^1^ The Barts Heart Centre, St Bartholomew's Hospital, Barts Health NHS Trust London UK

**Keywords:** atrial fibrillation, catheter ablation, coronary sinus, drivers, mapping

## Abstract

**Introduction:**

Markers predicting atrial fibrillation (AF) termination and freedom from AF/atrial tachycardia (AT) has been proposed. This study aimed to evaluate the role of novel coronary sinus (CS) electrogram characteristics in predicting the acute ablation response and freedom from AF/AT during follow‐up.

**Methods:**

Patients undergoing ablation for persistent AF as part of the Stochastic Trajectory Analysis of Ranked signals mapping study were included. Novel CS electrogram characteristics including CS cycle length variability (CLV) and CS activation pattern stability (APS) and proportion of low voltage zones (LVZs) were reviewed as potential predictors for AF termination on ablation and freedom from AF/AT during follow‐up. The relationship between localized driver characteristics and CS electrogram characteristics was also assessed.

**Results:**

Sixty‐five patients were included. AF termination was achieved in 51 patients and 80% of patients were free from AF/AT during a follow‐up of 29.5 ± 3.7 months. CS CLV of <30 ms, CS APS of ≥30% and proportion of LVZ < 30% showed high diagnostic accuracy in predicting AF termination on ablation and freedom from AF/AT during follow‐up (CS CLV odds ratio [OR] 25.6, area under the curve [AUC] 0.91; CS APS OR 15.9, AUC 0.94; proportion of LVZs OR 21.4, AUC 0.88). These markers were independent predictors of AF termination on ablation and AF/AT recurrence during follow‐up. Ablation of a smaller number of drivers that demonstrate greater dominance strongly correlate with greater CS organization.

**Conclusion:**

Novel CS electrogram characteristics were independent predictors of AF termination and AF/AT recurrence during follow‐up. These markers can potentially aid in predicting outcomes and guide ablation and follow‐up strategies.

## 1 INTRODUCTION

The success rate of catheter ablation for persistent atrial fibrillation (AF) remains suboptimal.^1^ Markers including left atrial (LA) size and AF duration have been utilized in predicting procedural success rate.^2^, ^3^ Achieving procedural AF termination has been linked to smaller LA size.^4^ Ablation of localized drivers has been utilized as a strategy for persistent AF ablation.^5^, ^6^, ^7^, ^8^ Localized drivers with certain electrogram characteristics such as lowest cycle length variability (CLV) and highest regularity index (RI) have shown to be associated with AF termination on ablation.^9^ The role of the coronary sinus (CS) electrogram characteristics in predicting AF termination and freedom from AF/atrial tachycardia (AT) during follow‐up has not yet been evaluated.

Stochastic Trajectory Analysis of Ranked Signals (STAR) is a novel‐mapping method for identifying localized drivers that potentially play a mechanistic role in maintaining AF. The STAR mapping method uses data on multiple individual wavefront trajectories to identify atrial regions that most often precede activation of neighboring areas with the aim of identifying intermittent drivers.^6^, ^10^ Pulmonary vein isolation (PVI) plus STAR mapping guided ablation using both global mapping with whole chamber basket catheters and sequential mapping with multipolar catheter has been associated with high rates of AF termination (76.3%) and high freedom from AF/AT (80%) during long‐term follow‐up.^6^, ^11^

The aim of the study was to determine whether novel CS electrogram characteristics can play a role in predicting AF termination on ablation and freedom from AF/AT during follow‐up. Prospective assessment of CS electrogram characteristics were performed in patients that underwent PVI plus STAR mapping guided ablation.

## 2 METHODS

### 2.1 STAR mapping principals

The STAR mapping method has been described in detail in previously published work.^5^, ^6^, ^10^ In brief, the principle of STAR mapping is to use data from multiple individual wavefront trajectories to identify regions of the atrium that most often precede activation of neighboring areas. By gathering data from many hundreds of activities, a statistical model can be formed. These permits regions of the atrium to be ranked according to the amount of time that activations precede those of adjacent regions (Online Supporting Information Methods).

### 2.2 STAR mapping cohort

#### 2.2.1 Patient selection

Patients with persistent AF were included (AF duration <24 months and no previous AF ablation). AF duration was defined as the duration of continuous AF in months i.e., the duration the patient has been in persistent AF. The only exclusion criteria for the study were inability to provide informed consent or having had a previous left atrial ablation. Patients were recruited consecutively. All patients provided informed consent for their study participation. The study complied with the Declaration of Helsinki and ethical approval was granted by the UK National Research Ethics System (16/LO/1379). The study was prospectively registered on clinicaltrials.gov (NCT02950844).

#### 2.2.2 STAR mapping ablation

The ablation protocol and STAR mapping unipolar recordings have been detailed in previous publications.^5^, ^6^ In brief, all patients underwent PVI with wide‐area circumferential ablation (WACA) and if AF remained post‐PVI, STAR maps were created using unipolar recordings obtained either with a whole‐chamber basket catheter or sequentially with multipolar catheters. AF drivers (AFDs) were targeted based on the STAR maps, 20‐min post‐PVI to ensure no discernable effect of PVI could affect rhythm or cycle length (CL) during AFD ablation. A study‐defined ablation response was either AF termination (AF termination to AT or AF termination to sinus rhythm (SR)) or CL slowing of ≥30 ms. If AF terminated before other AFDs had been ablated, these sites were not empirically targeted. Beyond targeting AFDs, no other empirical ablation was allowed including the creation of lines. If AF was organized into an AT this was mapped and ablated during the procedure. DC cardioversion was performed at the end of the procedure if AF did not terminate following ablation of all identified AFDs (Online Supporting Information Methods).

### 2.3 Predictors of AF termination on ablation

#### 2.3.1 CS electrogram characteristics

Unipolar electrogram recordings over 5 min from a deca catheter positioned in the CS were utilized for the analysis. The unipolar electrograms were recorded prospectively. The analysis of CS electrogram characteristics obtained for each patient was blinded to the ablation response and procedural outcomes until the end of the study. The unipolar electrograms were obtained by referencing to the ablation catheter positioned in the IVC. Unipolar signals were filtered with a bandpass filter of 0.5–500 Hz. The electrogram characteristics evaluated were CS CLV and CS activation pattern stability (APS). These markers were used to allow assessment of the level of CS organization with regard to both CL and activation pattern. The method used to establish the CS CLV and CS APS is detailed in online supporting information methods, Figures S2 and S3.

The relationship between both CS CLV and CS APS with AF termination as the response to ablation was assessed. The relationship between AFD characteristics and CS electrogram characteristics was also assessed. The localized driver characteristics evaluated included temporal stability and recurrence rate. Temporal stability was defined as the number of consecutive atrial activations during which the AFD was leading surrounding electrodes. Recurrence rate was defined as the proportion of time the AFD was leading surrounding electrodes during the duration of the recording.

#### 2.3.2 Bipolar voltage

All patients underwent a detailed bipolar voltage map created with the multipolar mapping catheter. The voltages obtained in the PVs were excluded. The color fill was set at 5 mm, and low voltage zones (LVZs) were defined as areas with a voltage of <0.2 mV. The relationship between the proportion of the left atrium (LA) that was LVZs and achieving AF termination with ablation was determined.

#### 2.3.3 Other potential predictive markers

LA size was determined in all patients and defined as the LA diameter obtained from the parasternal view on a transthoracic echocardiogram. Any underlying structural heart disease was also noted. These additional parameters were included in a multivariate analysis to determine which makers were potential predictive markers of AF termination on ablation.

### 2.4 Predictors of freedom from AF/AT

All patients in the STAR‐guided ablation cohort underwent clinical follow‐up at 3, 6, and 12 months, with 48‐h ambulatory Holter monitoring at 6 and 12 months. Patients were followed up six monthly thereafter. A 3‐month “blanking period” was observed, with all medication including antiarrhythmic drugs continued during this time. Clinical success was defined as freedom from AF/AT lasting >30 s off antiarrhythmic drugs subsequent to the 3‐month blanking period after a single procedure, as per consensus recommendations.^12^ The role of CS electrogram characteristics in predicting freedom from AF/AT was evaluated. The association with the proportion of the LA that was LVZs with freedom from AF/AT was also evaluated.

### 2.5 Statistical analysis

Statistical analyses were performed using SPSS (IBM SPSS Statistics, Version 24 IBM Corp.) and are detailed in Online Supporting Information Methods.

## 3 RESULTS

### 3.1 Baseline characteristics

Sixty‐five patients were included in the STAR mapping study (Figure S1). Baseline characteristics and general procedural and follow‐up data are summarized in Table S1. The average AF duration was 14.3 ± 5.4 months. One patient experienced cardiac tamponade that was noted at the end of the procedure that required pericardiocentesis.

### 3.2 Predictors of AF termination on ablation

Figure S1 demonstrates AF termination on a per patient and driver basis. The mean AF CL at baseline was 148 ± 5.6 ms and 151 ± 6.3 ms as measured in the CS and LAA, respectively. The average RF time post‐PVI to achieve AF termination was 6.5 ± 2.1 min. The AFDs demonstrated an average proportion of time leading of 86.6 ± 14.3%, that is, the electrode was leading its surrounding electrodes an average proportion of time of 86.6 ± 14.3%. The average number of consecutive atrial activations of which it was leading was 5.3 ± 0.4 per driver occurrence. AFDs, where AF termination occurred with ablation, showed a greater temporal stability (i.e., a higher number of consecutive atrial activations when leading surrounding electrodes) compared with drivers where CL slowing of ≥30 ms or no pre‐defined ablation response occurred on ablation (5.8 ± 0.5 vs. 4.3 ± 0.6; *p* = .002). They also showed a higher proportion of time leading surrounding electrodes compared to drivers where CL slowing of ≥30 ms or no pre‐defined ablation response occurred on ablation (79.5 ± 10.4% vs. 94.5 ± 15.6%; *p* = .006).

There was a significant correlation between CS CLV and CS APS (R of −0.84; *p* < .001). Therefore in the presence of greater CS APS the CS CLV was lower. Patients with a lower proportion of LVZs were more likely to have a lower CS CLV (*R* = 0.77, *p* < .001). Patients with a lower proportion of LVZs were also more likely to have a higher proportion of CS APS (*R* = −0.73, *p* < .001). When assessing the relationship between AF duration and CS electrogram characteristics there was no strong correlation between AF duration and CS CLV (*R* = 0.24, *p* = .06) or CS APS (*R* = 0.24, *p* = .06). AF duration also did not show a correlation with the proportion of LVZs whereby the longer duration of AF did not significantly correlate with a higher proportion of LVZs (*R* = −0.19, *p* = .13).

#### 3.2.1 CS electrogram characteristics

Patients with and without AF termination on ablation had a significantly different CS CLV (26.1 ± 4.6 ms vs. 41.9 ± 10.2 ms; *p* < .001) (Table 1). CS CLV was highly predictive of AF termination on ablation with an AUC of 0.85 (95% CI 0.70–1.01; *p* < .001). The optimal cutoff for CS CLV was <30 ms which had a sensitivity and specificity of 83.0% (95% CI 70.2–91.9) and 83.3% (95% CI 51.5–97.9) in predicting AF termination on ablation respectively (Table 2). The odds ratio of AF termination on ablation in a patient with a CS CLV of <30 ms was 24.4 (95% CI 4.6–131.0; *p* < .001).

**Table 1:** Demonstrates the differences in potential predictors between the group with AF termination on ablation versus the group without AF termination on ablation

Potential predictors	AF termination on ablation *n* = 51	No AF termination on ablation *n* = 14	*p* value
CS CLV ms mean ± SD	26.1 ± 4.6	41.9 ± 10.2	<.001
CS activation pattern stability % mean ± SD	44.4 ± 6.6	26.5 ± 8.0	<.001
Proportion of LVZs % mean ± SD	22.9 ± 5.6	43.5 ± 9.9	<.001
AF duration months mean ± SD	14.4 ± 5.2	11.7 ± 5.3	.62
LA size cm mean ± SD	4.1 ± 0.4	3.9 ± 0.4	.12
Antiarrhythmic drugs *n* (%)	36 (70.6)	9 (64.3)	.75
Age years mean± SD	61.4 ± 8.2	59.6 ± 11.8	.28
Male *n* (%)	39 (76.5)	8 (57.1)	.18
Previous cardiac surgery *n* (%)	1 (2.0)	0 (0)	1.00
Structural heart disease *n* (%)	3 (5.9)	1 (7.1)	1.00
CVA *n* (%)	1 (2.0)	1 (7.1)	.39
Hypertension *n* (%)	13 (25.5)	3 (21.4)	1.00

Abbreviations: AF, atrial fibrillation; CLV, cycle length variability; CVA, cerebrovascular accident; CS, coronary sinus; LVZs, low voltage zones; SD, standard deviation.

**Table 2:** Demonstrates the predictive value of these parameters with regard to predicting AF termination on ablation

Potential predictors	Sensitivity % (95% CI)	Specificity % (95% CI)	Positive predictive value % (95% CI)	Negative predictive value % (95% CI)
CS CLV < 30 ms	83.0 (70.2–91.9)	83.3 (51.5–97.9)	95.7 (86.1–98.7)	52.6 (36.8–68.0)
CS activation pattern stability ≥30%	90.7 (79.7–96.9)	72.7 (39.0–94.0)	94.2 (86.1–97.7)	61.5 (39.2–79.9)
Proportion of LVZs <30%	85.2 (73.9–93.4)	81.8 (48.2–97.7)	95.8 (86.7–98.8)	52.9 (35.9–69.3)
AF duration <12 months	33.3 (21.1–47.5)	54.6 (23.4–83.3)	83.1 (71.7–91.2)	14.3 (8.6–22.8)
LA size <4 cm	44.4 (30.9–58.6)	45.5 (16.8–76.6)	83.1 (71.7–91.2)	80.0 (68.4–74)
Antiarrhythmic drugs	72.2 (58.4–83.5)	45.5 (16.8–76.6)	86.7 (78.7–92.0)	25.0 (13.3–42.0)

Abbreviations: AF, atrial fibrillation; CI, confidence interval; CLV, cycle length variability; CS, coronary sinus; LVZs, low voltage zones.

CS APS was also shown to be significantly different between patients in whom ablation resulted in AF termination versus those it did not (44.4 ± 6.6% vs. 26.5 ± 8.0%; *p* < .001) (Table 1). CS APS was highly predictive of AF termination with ablation with an AUC of 0.86 (95% CI 0.82–0.94; *p* < .001). The optimal cutoff for CS APS was ≥30% which showed high sensitivity and specificity in predicting AF termination on ablation (Table 2). The odds ratio of AF termination on ablation with a CS APS of ≥30% was 26.1 (95% CI 5.2–41.4; *p* < .001).

**Figure 1:**
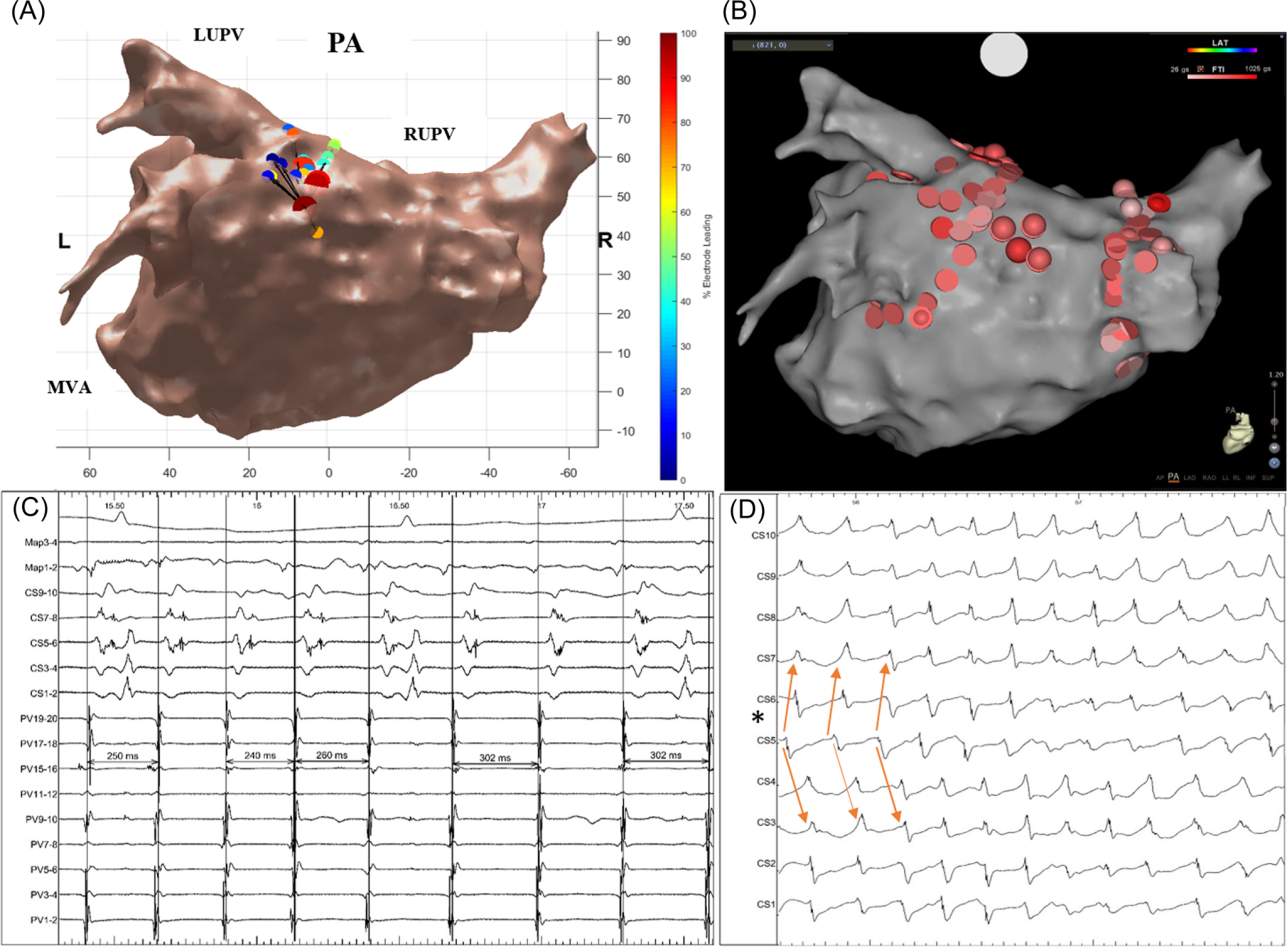
(A–D) Demonstrates (A) STAR map in a posterior‐anterior view that shows an AFD on the high posterior wall. (B) Ablation here as shown on the CARTO map in a posterior‐anterior view resulted in AF termination to an AT as shown on the (C) BARD electrograms including PV signals, CS signals, ablation catheter signals (Map), and surface ECG. (D) BARD electrograms demonstrating a proportion of the unipolar CS signals obtained during the 5‐min recording post‐PVI. The electrograms demonstrate a stable CS activation pattern whereby CS 5 (highlighted by a star) is leading its neighboring electrodes as shown by the orange arrows. Throughout the 5‐min recording, this activation pattern was present 42% of the time, i.e., the activation pattern stability was 42%. As shown on the CS electrograms there was minimal CL variation within the CS with a CLV of 21 ms during the 5‐min recording. AF, atrial fibrillation; CLV, cycle length variability; CS, coronary sinus; ECG, electrocardiogram; PVI, pulmonary vein isolation

**Figure 2:**
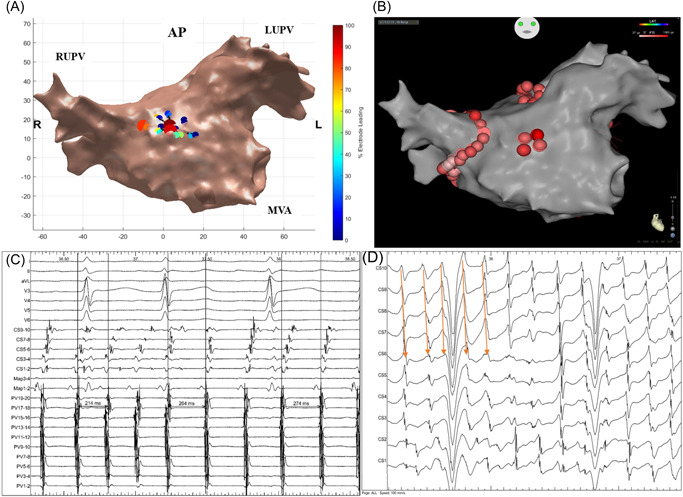
(A–D) Demonstrates (A) STAR map in an anterior‐posterior view that shows an AFD on the mid anterior wall. (B) Ablation here as shown on the CARTO map in an anterior‐posterior view resulted in AF termination to an AT as shown on the (C) BARD electrograms including PV signals, CS signals, ablation catheter signals (Map), and surface ECG. (D) BARD electrograms demonstrating a proportion of the unipolar CS signals obtained during the 5‐minute recording post‐PVI. The electrograms demonstrate a stable CS activation pattern whereby CS 10 (highlighted by a star) is leading its neighboring electrodes as shown by the orange arrows. Throughout the 5‐min recording, this activation pattern was present for 45% of the time, i.e., the activation pattern stability was 45%. As shown on the electrograms there was minimal CL variation within the CS with a CLV of 19.9 ms during the 5‐min recording. CLV, cycle length variability; CS, coronary sinus; ECG, electrocardiogram; PVI, pulmonary vein isolation

Due to the correlation between CS CLV, CS APS, and proportion of LVZs the binary logistic regression analysis was performed separately with each of these markers. This was to avoid multicollinearity biasing the results. CS CLV and CS APS were both shown to be independent predictors of AF termination on ablation (Table 3, Figures 1A–D and 2A–D).

**Table 3:** Binary logistic regression findings

	Univariate analysis		Multivariate analysis	
Potential predictors	*p* value	Odds ratio (95% CI)	*p* value	Odds ratio (95% CI)
**CS CLV model**				
CS CLV	<.001	0.79 (0.70–0.89)	.002	1.28 (0.67–1.90)
AF duration	.13	1.10 (0.97–1.27)	–	–
LA size	.24	2.74 (0.52–14.49)	–	–
Antiarrhythmic drugs	.25	2.17 (0.57–8.17)	–	–
Age	.55	1.02 (0.95–1.09)	–	–
Male	.16	0.22 (0.03–1.84)	–	–
Previous cardiac surgery	.25	0.19 (0.01–3.27)	–	–
Structural heart disease	.66	0.59 (0.06–6.25)	–	–
CVA	.89	0.23 (0.07–3.45)	–	–
Hypertension	.82	0.85 (0.20–3.66)	–	–
**CS activation pattern stability model**				
CS activation pattern stability	<.001	1.23 (1.10–1.37)	.009	1.39 (1.08–1.78)
AF duration	.13	1.10 (0.97–1.27)	–	–
LA size	.24	2.74 (0.52–14.49)	–	–
Antiarrhythmic drugs	.25	2.17 (0.57–8.17)	–	–
Age	.55	1.02 (0.95–1.09)	–	–
Male	.16	0.22 (0.03–1.84)	–	–
Previous cardiac surgery	.25	0.19 (0.01–3.27)	–	–
Structural heart disease	.66	0.59 (0.06–6.25)	–	–
CVA	.89	0.23 (0.07–3.45)	–	–
Hypertension	.82	0.85 (0.20–3.66)	–	–
**Proportion of LVZs model**				
Proportion of LVZs	<.001	0.89 (0.84–0.94)	.002	0.76 (0.64–0.90)
AF duration	.13	1.10 (0.97–1.27)	–	–
LA size	.24	2.74 (0.52–14.49)	–	–
Antiarrhythmic drugs	.25	2.17 (0.57–8.17)	–	–
Age	.55	1.02 (0.95–1.09)	–	–
Male	.16	0.22 (0.03–1.84)	–	–
Previous cardiac surgery	.25	0.19 (0.01–3.27)	–	–
Structural heart disease	.66	0.59 (0.06–6.25)	–	–
CVA	.89	0.23 (0.07–3.45)	–	–
Hypertension	.82	0.85 (0.20–3.66)	–	–

*Note*: CS CLV, CS activation pattern, and proportion of LVZs were incorporated separately with the other assessed parameters and were shown to be independent predictors of AF termination on ablation.

Abbreviations: AF, atrial fibrillation; CI, confidence interval; CLV, cycle length variability; CS, coronary sinus; CVA, cerebrovascular accident; LVZs, low voltage zones.

AFD characteristics were also shown to correlate to CS electrogram characteritics. The average temporal stability of the drivers targeted with ablation in each patient correlated with CS CLV (*R* = −0.74. *p* = .003) and CS APS (*R* = 0.79, *p* < .001). The average recurrence rate of the drivers targeted with ablation in each patient was also shown to correlate with CS CLV (*R* = −0.72, *p* = .002) and CS APS (*R* = 0.75, *p* < .001). There was also a correlation between the number of AFD ablated and the CS CLV (*R* = 0.75, *p* < .001) and CS APS (*R* = −0.73, *p* < .001) whereby in patients where a lower number of AFD ablated the CS CLV was lower and and the CS APS was higher.

Patients with a CS CLV of <30 ms were more likely to have drivers with higher temporal stability (5.9 ± 0.4 vs. 4.1 ± 0.5; *p* < .001) and recurrence rate (80.5 ± 9.2% vs. 95.6 ± 13.4%; *p* < .001) than those patients with a CS CLV of >30 ms. These findings also applied to CS APS whereby patients with a CS APS of ≥30% were more likely to have drivers with higher temporal stability (6.0 ± 0.5 vs. 4.0 ± 0.7; *p* < .001) and recurrence rate (81.5 ± 11.2% vs. 96.2 ± 16.1%; *p* = .006) than those patients with a CS APS of <30%. The number of drivers ablated was also significantly lower in patients with a CS CLV of <30ms compared to those with a CS CLV or >30 ms (2.1 ± 0.6 vs. 3.0 ± 0.8; *p* = .01). This was also true for a CS APS of ≥30% (2.3 ± 0.5 vs. 3.1 ± 0.8; *p* = .01).

#### 3.2.2 Proportion of LVZs

There was a significant difference in the proportion of LVZs in those patients in whom AF termination was achieved on ablation and those patients it did not (22.9 ± 5.6% vs. 43.5 ± 9.9%; *p* < .001) (Table 1). This was highly predictive of AF termination on ablation with an AUC of 0.85 (95% CI 0.81–0.92; *p* < .001). The optimal cutoff was <30% which showed a high sensitivity and specificity (Table 2). A proportion of LVZs of <30% showed an odds ratio of 37.7 (95% CI 6.9–52.5; *p* < .001) for predicting termination of AF with ablation. On multivariate analysis, the proportion of LVZs was an independent predictor of AF termination on ablation (Table 3).

#### 3.2.3 Other markers

The other makers assessed as per Table 1 including AF duration and LA size were not significantly different between patients in whom AF termination was achieved on ablation versus those it did not. Also, these makers showed no significant odds ratios in predicting AF termination on ablation (Table S2). AF duration of <12 months, LA size <4 cm and being on antiarrhythmic drugs had a low sensitivity and specificity in predicting AF termination on ablation (Table 2). They were also not independent predictors of AF termination (Table 3). There was no difference in the AF termination rates between patients with AF duration <12 months compared to those with an AF duration between 12 and 24 months (*p* = .56).

### 3.3 Predictors of freedom from AF/AT during follow‐up

The average follow‐up was 29.5 ± 3.7 months. Out of the 65 patients, 52 (80%) were free from AF/AT during follow‐up. Of the 13 patients in whom AF/AT reoccurred during follow‐up, four had a recurrence of AF and nine had recurrence of AT. Patients in whom AF termination was achieved with ablation were more likely to be free from AF/AT during follow‐up compared to those patients in whom AF termination was not achieved with ablation (48/51 vs. 4/14; *p* < .001). AF termination showed an odds ratio of 40.0 (95% CI 7.7–207.2; *p* < .001) for predicting freedom from AF/AT during follow‐up. On Kaplan‐Meier analysis, the survival free from AF/AT was significantly higher in patients in whom AF termination was achieved with ablation compared to those in whom AF termination was not achieved with ablation (log rank test *p* < .001).

#### 3.3.1 CS electrogram characteristics

Patients with and without AF/AT recurrence during follow‐up had a significantly different CS CLV (41.9 ± 7.7 ms vs. 25.4 ± 4.0 ms; *p* < .001) (Table 4) with CS CLV being lower in patients without AF/AT recurrence during follow‐up. CS CLV showed a high diagnostic accuracy in predicting freedom from AF/AT during follow‐up with an AUC of 0.91 (95% CI 0.82–1.00; *p* < .001). The optimal cutoff for CS CLV was <30 ms which had a sensitivity and specificity of 88.5% (95% CI 76.6–95.7) and 76.9% (95% CI 46.2–95.0) in predicting AF termination on ablation respectively. The positive predictive and negative predictive value was 93.9% (95% CI 85.0–97.7) and 66.7% (95% CI 48.1–81.2), respectively. The odds ratio of freedom from AF/AT during follow‐up with a CS CLV of <30 ms was 25.6 (95% CI 5.4–119.9; *p* < .001).

**Table 4:** Demonstrates the differences in potential predictors between the group with no AF/AT recurrence during follow‐up versus the group with AF/AT recurrence during follow‐up

Potential predictors	No AF/AT recurrence *n* = 52	AF/AT recurrence *n* = 13	*p* value
CS CLV ms mean ± SD	25.4 ± 4.0	41.9 ± 7.7	<.001
CS activation pattern stability % mean ± SD	45.6 ± 5.5	26.1 ± 7.2	<.001
Proportion of LVZs % mean ± SD	22.1 ± 5.8	42.6 ± 10.8	<.001
AF duration months mean ± SD	13.9 ± 5.4	14.4 ± 4.8	.38
LA size cm mean ± SD	4.1 ± 0.4	4.0 ± 0.4	.45
Antiarrhythmic drugs *n* (%)	39 (75.0)	7 (53.8)	.18
Age years mean± SD	61.4 ± 9.0	59.9 ± 8.6	.30
Male *n* (%)	38 (73.1)	9 (69.2)	.74
Previous cardiac surgery *n* (%)	1 (1.9)	0 (0)	1.00
Structural heart disease *n* (%)	3 (5.8)	1 (7.1)	1.00
CVA *n* (%)	2 (3.8)	0 (0)	1.00
Hypertension *n* (%)	14 (26.9)	2 (15.4)	.49

Abbreviations: AF, atrial fibrillation; AT, atrial tachycardia; CLV, cycle length variability; CVA, cerebrovascular accident; CS, coronary sinus; LVZs, low voltage zones; SD, standard deviation.

CS APS was also shown to be significantly different between patients with and without AF/AT recurrence during follow‐up (26.1 ± 7.2% vs. 45.6 ± 5.5%; *p* < .001) (Table 4). CS APS showed a high degree of accuracy in predicting freedom from AF/AT during follow‐up with an AUC of 0.94 (95% CI 0.88–1.00; *p* < .001). The optimal cutoff for CS APS was ≥30% which had a sensitivity and specificity of 93.5% (95% CI 82.1–98.6) and 52.6% (95% CI 28.9–75.6), respectively. The positive predictive and negative predictive value was 82.7% (95% CI 74.7–88.5) and 76.9% (95% CI 50.7–91.5), respectively. The odds ratio of freedom from AF/AT during follow‐up with a CS APS of ≥30% was 15.9 (95% CI 3.6–69.7; *p* < .001).

Due to the correlation between CS CLV, CS APS and proportion of LVZs, Cox regression hazard ratio was again performed seperately with each of these markers. This was to avoid multicollinearity biasing the results. CS CLV and CS APS were shown to be independent predictors of AF/AT recurrence during follow‐up (Table 5).

**Table 5:** Demonstrates the Cox proportional regression analysis in regard to freedom from AF/AT during follow‐up

	Univariate analysis		Multivariate analysis	
	*p* value	Hazards ratio (95% CI)	*p* value	Hazards ratio (95% CI)
**CS CLV model**				
CS CLV	<.001	1.15 (1.09–1.22)	<.001	1.17 (1.09–1.26)
AF duration	.72	1.02 (0.92–1.13)	–	–
LA size	.90	0.92 (0.36–3.29)	–	–
Antiarrhythmic drugs	.94	0.96 (0.39–3.11)	–	–
Age	.57	0.98 (0.93–1.04)	–	–
Male	.85	0.89 (0.28–2.90)	–	–
Previous cardiac surgery	.75	0.05 (0.01–1.27)	–	–
Structural heart disease	.52	0.61 (0.09–5.25)	–	–
CVA	.67	0.05 (0.01–1.45)	–	–
Hypertension	.44	0.55 (0.12–2.50)	–	–
**CS activation pattern stability model**				
CS activation pattern stability	<.001	0.84 (0.78–0.92)	<.001	0.84 (0.78–0.91)
AF duration	.72	1.02 (0.92–1.13)	–	–
LA size	.90	0.92 (0.36–3.29)	–	–
Antiarrhythmic drugs	.94	0.96 (0.39–3.11)	–	–
Age	.57	0.98 (0.93–1.04)	–	–
Male	.85	0.89 (0.28–2.90)	–	–
Previous cardiac surgery	.75	0.05 (0.01–1.27)	–	–
Structural heart disease	.52	0.61 (0.09–5.25)	–	–
CVA	.67	0.05 (0.01–1.45)	–	–
Hypertension	.44	0.55 (0.12–2.50)	–	–
**Proportion of LVZs model**				
Proportion of LVZs	<.001	1.15 (1.09–1.21)	<.001	1.22 (1.12–1.32)
AF duration	.72	1.02 (0.92–1.13)	–	–
LA size	.90	0.92 (0.36–3.29)	–	–
Antiarrhythmic drugs	.94	0.96 (0.39–3.11)	–	–
Age	.57	0.98 (0.93–1.04)	–	–
Male	.85	0.89 (0.28–2.90)	–	–
Previous cardiac surgery	.75	0.05 (0.01–1.27)	–	–
Structural heart disease	.52	0.61 (0.09–5.25)	–	–
CVA	.67	0.05 (0.01–1.45)	–	–
Hypertension	.44	0.55 (0.12–2.50)	–	–

Abbreviations: AF, atrial fibrillation; AT, atrial tachycardia; CI, confidence interval; CLV, cycle length variability; CS, coronary sinus; CVA, cerebrovascular accident; LVZs, low voltage zones.

**Figure 3:**
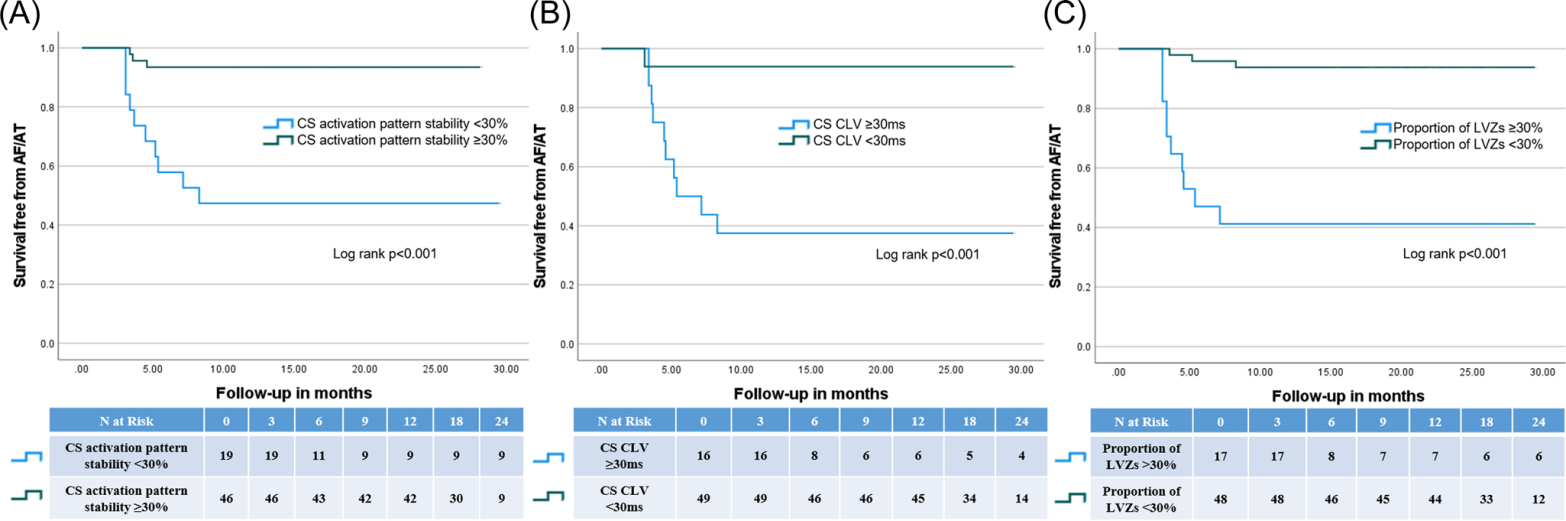
(A–C) Demonstrates Kaplan–Meier curves that show a significant difference in survival free from AF/AT in patients based on differences in (A) CS activation pattern stability whereby patients with a CS activation pattern stability of ≥30% have an increase survival free from AF/AT compared with patients with a CS activation pattern stability of <30%. (B) CS CLV whereby patients with a CS CLV of <30 ms have an increase survival free from AF/AT compared with patients with a CS CLV of ≥30 ms. (C) Proportion of LVZs whereby patients with a proportion of LVZs of <30% have an increased survival free from AF/AT compared to patients with a proportion of LVZs of ≥30%. AF, atrial fibrillation; AT, atrial tachycardia; CLV, cycle length variability; CS, coronary sinus; LVZs, low voltage zones

Survival free from AF/AT was significantly higher in patients with a CS APS of ≥30% and CS CLV < 30 ms (Figure 3A–C).

#### 3.3.2 Proportion of LVZs

There was a significant difference in the proportion of LVZs in those patients with AF recurrence and those without (42.6 ± 10.8 vs. 22.1 ± 5.8; *p* < .001) (Table 4). This showed a high diagnostic accuracy in predicting freedom from AF/AT during follow‐up with an AUC of 0.88 (95% CI 0.76–1.00; *p* < .001). The optimal cutoff was <30% which showed a sensitivity and specificity of 93.8% (95% CI 82.8–98.7) and 58.8% (95% CI 32.9–81.6), respectively. Positive and negative predictive value was 86.5% (95% CI 78.4–91.9) and 76.9% (95% CI 51.0–91.5). A proportion of LVZs of <30% showed an odds ratio of 21.4 (95% CI 4.7–97.6; *p* < .001) in predicting freedom from AF/AT during follow‐up. Proportion of LVZs was shown to be an independent predictor of AF/AT recurrence during follow‐up (Table 5). Survival free from AF/AT was significantly higher in patients with a proportion of LVZ <30% (Figure 3A–C).

#### 3.3.3 Other markers

The other makers assessed as per Table 4 including AF duration and LA size were not significantly different between patients in whom there was AF/AT recurrence during follow‐up compared to those that did not have AF/AT recurrence during follow‐up. None of these markers were shown to be independent predictors of AF/AT recurrence during follow‐up (Table 5). There was no difference in the AF/AT recurrence rate between patients with an AF duration between <12 months and those with an AF duration between 12 and 24 months (*p* = .67).

## 4 DISCUSSION

This study demonstrates that novel CS electrogram characteristics including CS CLV and CS APS, in addition to the proportion of LVZs are independent predictors of achieving AF termination on ablation and freedom from AF/AT during long‐term follow‐up with a high degree of accuracy in a cohort that is a good representation of a general persistent AF cohort (average AF duration of 14 months and LA area 32 cm^2^). These markers are far more effective in identifying those patients that are more likely to have successful outcomes from a persistent AF catheter ablation than markers such as LA size and AF duration that are currently widely used to predict procedural outcomes and may influence ablation strategies utilized. The study has also shown that the characteristics of CS electrogram correlate strongly with the proportion of LA LVZs, the number of localized drivers identified, and the temporal stability and recurrence rate of identified drivers.

### 4.1 CS electrogram organization and outcome

Previous studies have shown that drivers with lower CS CLV or higher RI are more likely to be associated with AF termination on ablation^9^, ^13^ highlighting the importance of electrogram organization in regard to mechanisms maintaining AF. This is further supported by the findings of this study whereby novel markers measuring the level of organization in the CS were shown to be independent predictors of AF termination with ablation and clinical success at long‐term follow‐up. This is the first study that has evaluated CS characteristics as a predictor of procedural outcomes. In this study, a CS CLV of <30 ms and CS activation pattern stability of ≥30% were associated with a high odds ratio in predicting AF termination on ablation and freedom from AF/AT during follow‐up. Both of these markers were strong independent predictors of AF termination and freedom from AF/AT during follow‐up with a significant AUC.

### 4.2 CS electrogram organization and driver properties

Greater CS organization was also associated with a smaller number of AFDs identified, greater driver temporal stability, and higher recurrence rate, which have previously been shown to be driver characteristics that are associated with AF termination on ablation.^9^ This finding is physiologically plausible in that a smaller number of drivers demonstrating greater dominance in terms of greater temporal stability and higher recurrence rate are more likely to activate the CS in a consistent manner than a larger number of less dominant drivers. This would explain why AF driver properties correspond to CS electrogram characteristics, why each has implications for AF mechanisms, and why both are linked to AF termination on ablation and clinical outcome.

### 4.3 AF termination as an endpoint

In this study, it was shown that AF termination on ablation strongly correlated with freedom from AF/AT during follow‐up. The role of AF termination on ablation in predicting procedural outcomes has shown conflicting evidence.^14^, ^15^ However, studies that have assessed the role of AF termination as a predictor of freedom from AF/AT have all been limited to a stepwise ablation strategy with CFAE and/or linear ablation.^16^ Further to this, it has been argued that the conflicting evidence regarding AF termination on ablation as a predictor of outcomes relies on the timing at which AF termination is achieved.

Studies in which AF termination has been an independent predictor of outcomes have arguably been where a majority of patients terminated early on during catheter ablation^1^, ^14^ and shorter ablation duration has shown to be an independent predictor of clinical success.^4^ In this study, the average duration of ablation post‐PVI was around 7 min with a total ablation duration during AF of around 60 min. This is compatible with the duration labeled as termination “early in the ablation procedure” making this study finding consistent with these previous studies that have shown that AF termination is a predictor of the clinical outcome if it occurs early on during the ablation. These findings further emphasize that ablation strategies incorporating driver mapping aiming to terminate AF with limited ablation are perhaps most likely to obtain the best clinical outcome.

### 4.4 Relationship between LVZ, CS organization, and other clinical factors

In this study, it was shown that there was no significant correlation between the proportion of LVZs and AF duration, which is supported by the findings of previous studies.^17^ Similarly, there was no correlation between LVZs and LA size or other risk factors for AF progression. CS electrogram characteristics also did not correlate with AF duration, LA size, or other risk factors for AF progression, whilst they did correlate significantly with the proportion of LA LVZs. These findings highlight several important points. First, AF duration, LA size, and other risk factors for AF progression do not predict the degree of LA structural and electrical remodeling in regard to bipolar voltage and the level of CS organization. Second, the level of LA remodeling based on bipolar voltage has a direct impact on the electrical characteristics in the LA. This has been supported by previous studies, which have shown that baseline CV, and CV dynamics in the LA differ depending on the underlying bipolar voltage.^18^ Further to this, the proportion of LVZs was shown in this study to be a strong independent predictor of AF termination and AF/AT recurrence during follow‐up whereby patients with a proportion of LVZs <30% were more likely to have AF termination on ablation and no AF/AT recurrence during follow‐up. These findings are supported by that of previous studies, which have shown that the presence of LA scarring is a predictor of AF recurrence.^17^

### 4.5 LVZs, CS organization and current categorization of AF

Currently, AF duration is the only parameter used to define AF types into paroxysmal, persistent, and longstanding persistent AF. However, in this study AF duration was not shown to be an independent predictor of either AF termination on ablation or AF/AT recurrence during follow‐up, which is supported by the findings of previous studies.^3^ Studies that have shown that AF duration is a predictor of clinical success have not adjusted for additional markers such as proportion of LVZs and CS electrogram characteristics that were shown to be independent predictors in this study.^2^ It is possible that the impact of LA size and AF duration observed in previous studies may have been driven by outliers in terms of patients with very long‐standing persistent AF or massively dilated atria who may no longer be selected for ablation. It is important to note that all patients in this study had AF durations of less than 24 months with an average AF duration of around 14 months, and atria were moderately dilated. Smaller differences in LA size and AF duration may be less relevant to the outcome. This study indicates that differences in AF duration below 24 months do not independently lead to differences in electrophysiological endpoints and procedural outcomes, although AF duration of greater than 24 months may still be relevant.

Currently, AF is defined as early persistent if the AF duration is <12 months whilst AF duration of >12 months is defined as longstanding persistent AF.^12^ Based on the findings from this study, there was no difference in AF termination and AF/AT recurrence rate between patients with an AF duration <12 months and patients with an AF duration of >12 months. Therefore, the current categorization of AF in this way is not useful prognostically. AF may be more usefully categorized using measures outlined here which are prognostically useful. Whilst the proportion of LA LVZs was similarly useful, this requires a mapping system, expensive mapping catheters, and time to acquire a voltage map. It is feasible that CS electrograms could provide equivalent data as a stand‐alone diagnostic test, or at the time of simple ablation using a single shot technology, or perhaps at the start of a case to plan the ablation strategy and yield prognostic procedural outcome data.

Studies have proposed that cardiac MRI can evaluate atrial scar and remodeling and show a strong correlation to the bipolar voltage maps obtained during catheter ablation.^19^ Again, it is possible that this data could be derived quickly and cheaply from a CS electrogram.

### 4.6 STAR mapping

CARTO has recently produced CARTOFINDER which times electrograms relative to each other in a 250 ms window which then moves through a continuous recording to show wavefront movement over time. This has enjoyed some success demonstrating focal and rotational activations in AF and an automated approach to their detection has been developed.^7^ The Topera and ECGI mapping systems rely on phase mapping to identify drivers. These technologies have also enjoyed some success; although there has also been concern that phase mapping could demonstrate rotors incorrectly at times.^20^ In contrast the STAR mapping method utilizes minimal computation or data manipulation and simply compares activation times across electrode pairs to identify sites that are most often leading compared with neighboring sites. Furthermore, all currently available mapping systems require a degree of interpretation by the operator, such as for analysis of phase maps with the ECGI, or analysis of dynamic wavefront activation maps with CARTOFINDER (Biosense Webster). In contrast, the STAR mapping method is not dependent on the analysis of dynamic wavefront maps but works through highlighting the ablation target sites by a colored circle (circle color being representative of the time an electrode is leading relative to its pairs). The STAR mapping methodology, therefore, has some potential advantages for mapping in AF and clinical studies have shown beneficial results.

### 4.7 Limitations

This study has several important limitations. First, the STAR mapping cohort consists of a relatively small number of patients. However, this cohort of 65 patients was thought reasonable as a mechanistic study using a novel mapping technique. The number of patients undergoing persistent AF ablation is similar to previously reported studies using a novel mapping system.^7^ Furthermore, all patients underwent driver ablation guided by STAR mapping and it is unclear whether these results apply to other contexts such as conventional ablation or PVI alone for AF, or simply cardioversion. Due to the high rate of AF termination and freedom from arrhythmia recurrence during follow‐up, the comparison cohort was smaller and as a result, the significance of the difference between these cohorts might have been underestimated.

## 5 CONCLUSIONS

Novel CS electrogram characteristics including CS CLV and CS APS and proportion of LVZs were shown to be strong independent predictors of AF termination on ablation and AF/AT recurrence during follow‐up. These three makers were far superior to AF duration and LA size in predicting outcome. Driver characteristics were also closely intertwined with CS electrogram characteristics whereby a smaller proportion of drivers with greater dominance were associated with greater CS organization. CS electrogram organization may be a surrogate for LVZs and therefore presumably CMR assessment of scar and may be useful in predicting outcome and planning ablation strategy. AF categorization based on the organization may be clinically more useful than current definitions with regard to outcomes.

## Supporting information

Supplementary information.

Supplementary information.

Supplementary information.

Supplementary information.

Supplementary information.

Supplementary information.

Supplementary information.

## Data Availability

The data underlying this article will be shared on reasonable request to the corresponding author.
