# Supplementary material for: Coronary sinus electrogram characteristics predict termination of AF with ablation and long‐term clinical outcome
Source: J Cardiovasc Electrophysiol. 2022 Jul 28;33(10):2139–51. doi: 10.1111/jce.15618 (PMC9796101; doi:10.1111/jce.15618)
Supplement: Supplementary file 1 — Supplementary information. [file JCE-33-2139-s005.docx]

**Supplemental Figure Legend**

***Supplemental Figure 1-*** *Consort flow diagram*

***Supplemental Figure 2-*** *Demonstrates a cycle length (CL) histogram obtained at one of the basket catheter electrodes, with percentage of recording made up by each CL on the y-axis and CL on the x-axis. Each bar represents a defined a CL. For illustrative purposes less frequent CLs along the mean were excluded. The dashed line (- -) demonstrates the narrowest range of CLs making up 50% of the cycles (under the taller bell shaped curve). This corresponds to the dominant CL which is highlighted as a red bar. The other dashed lines ( ) demonstrate the SD of CLs.*

***Supplemental Figure 3-*** *An illustration to aid in demonstrating how CS activation pattern stability was determined. The illustration shows atrial activations obtained from the 10 electrodes on the deca catheter within the CS. Six atrial activations are included. The first step in determining the CS activation pattern stability is to identify the overall leading electrode. During the 6 atrial activations CS 8 (highlighted by a *) is leading the other CS electrodes for 4 atrial activations. These atrial activations are highlighted by the red box. For 2 atrial activations again highlighted by the red box CS 5 (highlighted by a *) is leading the other CS electrodes Therefore the electrode that is leading the greatest proportion of time during the 6 atrial activations is CS 8. This is therefore deemed the overall leading electrode. Following this the activation pattern between CS 8 and its neighbouring 4 electrodes (CS6, 7, 9 and 10) is determined. There are 3 activation patterns identified: CS 8-CS 7-CS9-CS10-CS6 (orange arrows), CS8-CS9-CS10-CS7-CS6 (red arrows) and for the last atrial activation CS8 is not leading with a CS activation pattern of CS9-CS6-CS10-CS8-CS7. The first activation pattern occurred for 2 atrial activations (33%), the second one for 3 atrial activations (50%) and the last one for 1 atrial activation (17%). The median of the proportion of time each activation pattern was present is then taken to represent the CS activation pattern stability which in this example is 33%.*
