# Supplementary material for: Coronary sinus electrogram characteristics predict termination of AF with ablation and long‐term clinical outcome
Source: J Cardiovasc Electrophysiol. 2022 Jul 28;33(10):2139–51. doi: 10.1111/jce.15618 (PMC9796101; doi:10.1111/jce.15618)
Supplement: Supplementary file 2 — Supplementary information. [file JCE-33-2139-s007.docx]

**Supplemental Methods**

***STAR mapping method***

In brief, the principle of STAR mapping is to use data from multiple individual wavefront trajectories to identify regions of the atrium that most often precede activation of neighboring areas. By gathering data from many hundreds of activations, a statistical model can be formed. These permits regions of the atrium to be ranked according to the amount of time that activations precede those of adjacent regions. Electrodes are paired using pre-defined geodesic distances, the activation times are then compared between the electrodes in all pairs using all wavefront trajectories to establish a leading site and thereby the direction of wavefront propagation. For a leading site to be classified as an AF driver (AFD) it is required to lead at least 75% of the time. Unipolar activation times were taken at the peak negative dv/dt. The STAR mapping method filters ventricular far field signals. To exclude implausible wavefront trajectories the STAR mapping method filters wavefront activations based on plausible activation time differences established using geodesic distance and pre-defined conduction velocities (CVs). To avoid annotations on noise or fractionated electrograms, signals are filtered using the refractory period.

***STAR mapping ablation***

All procedures were performed using a 3D mapping system (CARTO Biosense Webster, Diamond Bar, MA, USA; Rhythmia, Boston Scientific, MA, USA and EnSite Precision system, Abbott, CA, USA). All patients underwent PVI with wide area circumferential ablation (WACA) using an irrigated radiofrequency (RF) ablation catheter. Twenty minutes following PVI further ablation in the LA was guided by the STAR maps created using imported unipolar electrogram signals, chamber geometry and catheter electrode location data that were processed by a Matlab custom written script (Matlab 2017b, MathWorks MA, USA). The STAR maps were created using unipolar recordings that were obtained post-PVI either using whole-chamber basket catheters (Constellation, Boston Scientific, Natick, MA or FIRMap Abbott, CA, USA) or pulmonary vein (PV) mapping catheters (PentaRay catheter with CARTO, IntellaMap Orion with Rhythmia and Advisor HD Grid with EnSite Precision System). Prospective studies have shown that both mapping modalities can effectively identify AFD ^7, 13, 14^. When using the basket catheters, a minimum of two recordings were taken in different positions if required to achieve optimal LA coverage. With PV mapping catheters a minimum of 10 recordings were taken to ensure optimal LA coverage. The STAR maps were used to identify AFD that were projected onto a replica of the geometry created in the 3D mapping system which allows the location of the AFD identified on the STAR map to be tagged on the geometry created with the 3D mapping system. All AFDs on a STAR map were targeted in order of ranked priority whereby sites that were leading 100% of the time were targeted first followed by those leading 90% of the time, etc. If multiple AFD with the same ranked priority were identified, it was at the operator’s discretion in what order to ablate the AFD.

Initial mapping and ablation were performed in the LA, and right atrium (RA) mapping was only performed if an AFD in the RA was considered likely (CS activation predominantly proximal to distal and fastest CL at the LA septum).

During ablation of AFD, a lesion was delivered at the centre of the driver site with further ablation surrounding the initial lesion in a cluster, avoiding the creation of linear lesions so as not to affect any AF mechanisms in this way. Ablation at driver sites was delivered with a contact force of 5-40 g, with a power of 30-40W (30W posteriorly and 40W elsewhere). Ablation at a driver site was stopped if: a total of 5 minutes of ablation had been performed at an AFD including consolidating ablation lesions, or no signal remained at the ablation site**,** or a study-defined ablation response had been achieved. A study-defined ablation response was either AF termination or cycle length (CL) slowing of ≥30ms. If AF terminated before other AFD had been ablated, these sites were not empirically targeted. Beyond targeting AFD, no other empirical ablation was allowed including the creation of lines. If AF organized into an AT this was mapped and ablated during the procedure. DC cardioversion was performed at the end of the procedure if AF did not terminate following ablation of all identified AFD.

***CS electrogram characteristics***

CS electrogram characteristics were evaluated using a customized automated script written in Matlab. The script has been developed and validated using 30 minutes of CS unipolar recordings which has effectively shown that all atrial activations in AF were effectively identified using this script. CS CLV was established by reviewing the CS CLs over a 5-minute recording. Each CL was taken as the CL between two consecutive atrial activations. Ventricular far field signals were filtered. To avoid annotations on noise or fractionated electrograms, signals were filtered using the refractory period. All the CL measurements over the 5-minute recording for each electrode was used to create a histogram of the CLs with all CLs identified on the x-axis (rounded to the nearest whole millisecond) and the percentage of recording made up by each CL on the y-axis were plotted for each individual electrode for each 5-minute recording for each patient. The dominant CL of all the CLs was then identified. The dominant CL was defined as the centre of the narrowest range of CLs in the histogram containing 50% of the cycles. The CLV was determined by taking the standard deviation (SD) of CLs (Supplemental Figure 1). A smaller CLV therefore denotes less CL variation and greater CS organization.

CS activation pattern stability was determined by assessing the CS activation pattern over a 5-minute recording using unipolar electrograms. The first step was to identify the overall leading electrode. This was achieved by determining for each atrial activation the electrode that was leading the other CS electrodes. The electrode that was then leading the greatest proportion of time taking into account all the atrial cycles during the 5-minute recording was defined as the overall leading electrode. Once the leading electrode had been identified, the activation pattern of the leading electrode relative to the four neighbouring electrodes was determined. For each atrial activation, the activation time differences were compared between the electrode defined as the leading electrode and its four neighbouring electrodes.

The activation time difference allows the order each neighbouring electrode is activated to be determined and thereby the activation pattern for each activation. To identify the activation pattern during cycles when the overall leading electrode is not leading, the activation time ascribed relative to the overall leading electrode was negative indicating that the overall leading electrode is following the comparison electrode during that cycle. All the different activation patterns were identified and the proportion each activation pattern occurred during the 5-minute recording was determined. The median of these proportions was taken as the CS activation pattern stability (Supplemental Figure 2).

***Statistical Analysis***

Continuous variables are displayed as mean ± standard deviation (SD) or median (interquartile range). Categorical variables are presented as a number and percentage. Chi-square was used for the comparison of nominal variables. The student t-test, or its non-parametric equivalent, Mann-Whitney U test when appropriate was used for comparison of continuous variables. P-value of <0.05 was deemed significant. To assess for the correlation between two variable, Pearson correlation coefficient was calculated. Area under the curve, sensitivity, specificity, positive and negative predictive values were calculated for potential predictors. Odds ratio was calculated for the optimal cutoffs identified for the potential predictors in regard to AF termination on ablation and freedom from AF/AT during follow-up. Binary logistic regression and Cox proportional hazard ratio was also performed to elicit independent predictors for AF termination on ablation and freedom from AF/AT respectively. Markers included in the analysis were CS CLV, CS activation pattern stability, proportion of LVZs, AF duration, LA size, previous cardiac surgery, presence of structural heart disease, previous cerebrovascular accident (CVA), known hypertension, age and male gender. Kaplan Meier curves were plotted to assess differences in survival free from AF/AT during follow-up.
