# Supplementary material for: Coronary sinus electrogram characteristics predict termination of AF with ablation and long‐term clinical outcome
Source: J Cardiovasc Electrophysiol. 2022 Jul 28;33(10):2139–51. doi: 10.1111/jce.15618 (PMC9796101; doi:10.1111/jce.15618)
Supplement: Supplementary file 3 — Supplementary information. [file JCE-33-2139-s006.docx]

***Supplemental Table 1****- Baseline characteristics*

| **Baseline characteristics** | | **STAR Cohort n=65** |
| --- | --- | --- |
| Age years mean ± SD | 60.9±9.4 | |
| Male n (%) | 47 (72.3) | |
| Anti-arrhythmic drugs n (%) | 46 (70.8) | |
| Hypertension n (%) | 16 (24.6) | |
| Diabetes mellitus n (%) | 0 (0) | |
| CVA^Ͳ^ n (%) | 2 (3.1) | |
| Structural heart disease n (%) | 4 (6.2) | |
| Previous cardiac surgery n (%) | 1 (1.5) | |
| LA area cm^2^ mean±SD | 32.0±0.8 | |
| AF duration months mean±SD | 14.3±5.4 | |
| **Procedural data**  General anaesthetic for procedure  AF termination with ablation  Procedural duration min mean±SD  Total ablation min mean±SD  Total ablation post PVI^Ŧ^ during AF mean±SD  Fluoroscopy time min mean±SD  Complications n (%) | 18 (27.7)  51 (78.5)  225.4±65.6  63.5±10.2  6.5±2.1  1.9±3.2  1 (1.5) | |
| **Follow-up data**  Follow-up months mean±SD  Freedom from AF/AT n (%) | 29.5±3.7  52 (80.0) | |

^Ͳ^CVA- Transient ischemic event/Cerebrovascular accident

^Ŧ^PVI- Pulmonary vein isolation
