# Supplementary material for: Coronary sinus electrogram characteristics predict termination of AF with ablation and long‐term clinical outcome
Source: J Cardiovasc Electrophysiol. 2022 Jul 28;33(10):2139–51. doi: 10.1111/jce.15618 (PMC9796101; doi:10.1111/jce.15618)
Supplement: Supplementary file 4 — Supplementary information. [file JCE-33-2139-s001.docx]

*Supplemental Table 2- Demonstrates the Odds ratio of potential markers predicting AF termination on ablation.*

| Potential predictors | Odds ratio  (95%CI) | P-value |
| --- | --- | --- |
| CS CLV <30ms | 24.4 (4.6-131.0) | <0.001 |
| CS activation pattern stability ≥30% | 26.1 (5.2-41.4) | <0.001 |
| Proportion of LVZs <30% | 37.7 (6.9-52.5) | <0.001 |
| AF duration ≤ 12months | 0.6 (0.2-2.2) | 0.45 |
| LA size <4cm | 0.67 (0.2-2.5) | 0.54 |
| Anti-arrhythmic drugs being taken | 2.2 (0.6-8.2) | 0.25 |
| Age >60 yrs. | 0.8 (0.2-3.1) | 0.75 |
| Being male | 2.4 (0.6-8.9) | 0.19 |
| Previous cardiac surgery | 0.2 (0.01-3.3) | 0.25 |
| Known structural heart disease | 0.6 (0.06-6.2) | 0.44 |
| Previous CVA | 0.6 (0.02-15.5) | 0.75 |
| Known Hypertension | 0.4 (0.1-1.5) | 0.16 |
