# Supplementary figures and images for: Coronary sinus electrogram characteristics predict termination of AF with ablation and long‐term clinical outcome
Source: J Cardiovasc Electrophysiol. 2022 Jul 28;33(10):2139–51. doi: 10.1111/jce.15618 (PMC9796101; doi:10.1111/jce.15618)

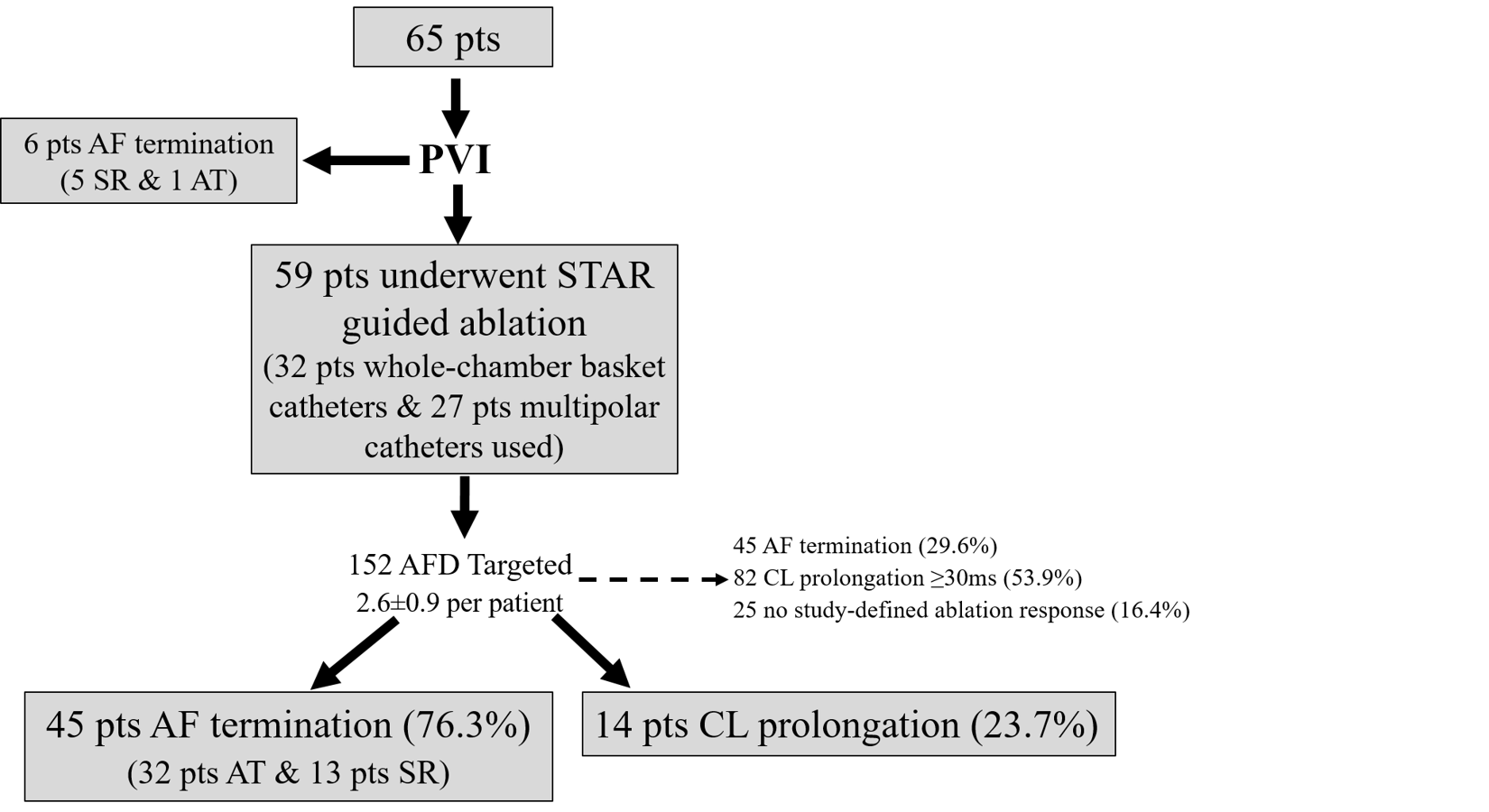

Supplement: Supplementary file 5 — Supplementary information. [file JCE-33-2139-s003.tif]

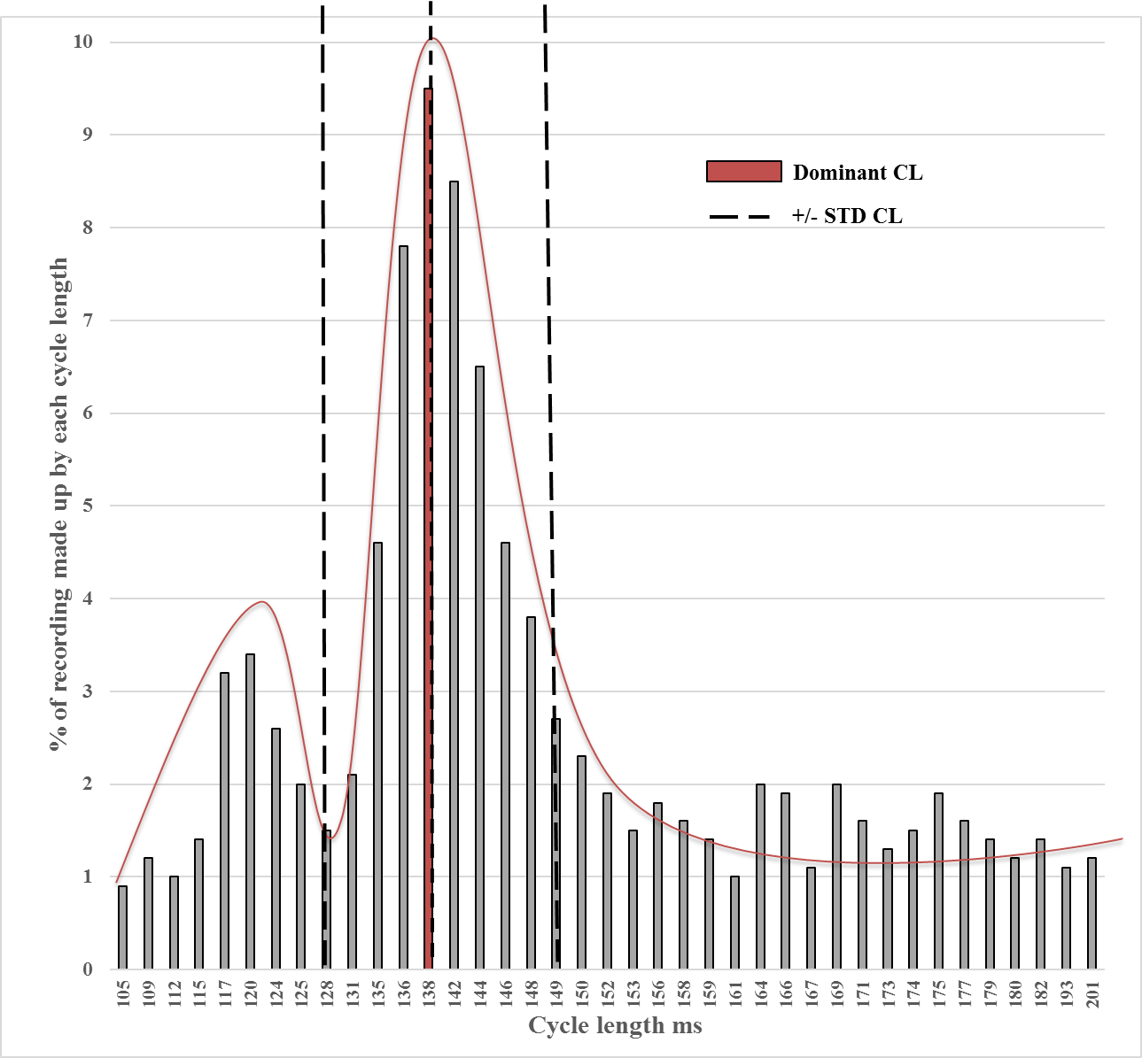

Supplement: Supplementary file 6 — Supplementary information. [file JCE-33-2139-s002.tif]

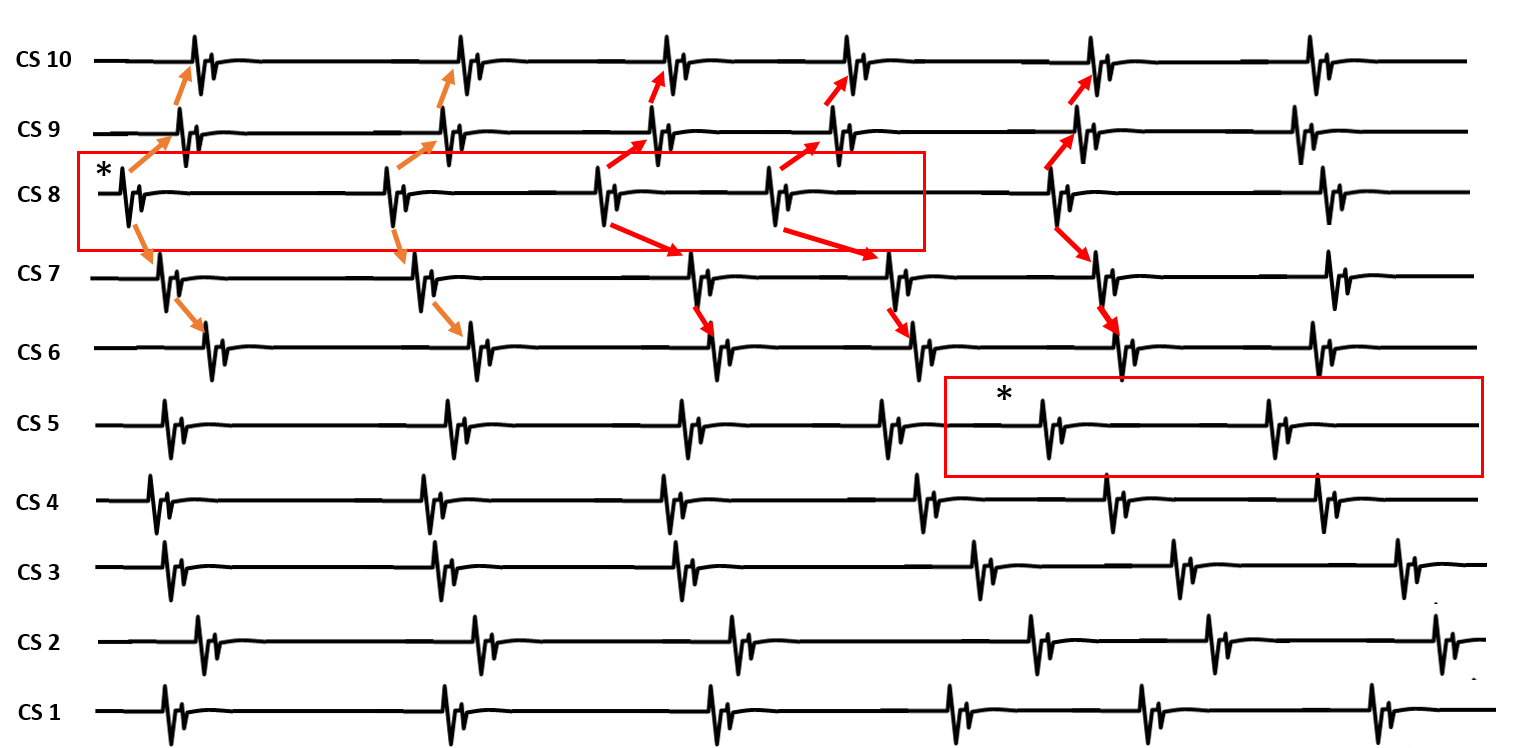

Supplement: Supplementary file 7 — Supplementary information. [file JCE-33-2139-s004.tif]
